# Philadelphia Correspondence

**Published:** 1868-07-15

**Authors:** 


### *PHILADELPHIA CORRESPONDENCE.*

PHILADELPHIA, June 20, 1868.

*To Chicago Medical Journal.*

I have had a very interesting case of burn produced by action of condensed lye, and intended reporting it for your columns, but will wait till next No., as it has not yet passed its dangerous point. I therefore resume the account of the clinical cases.

M. J., age 52.—Carcinoma of left mammary gland of eighteen months' standing. Elliptical incision made, and tumor removed. Parts united nicely, and patient discharged.

W. E., age 38.—Encephaloid involving the antrum of Highmore. Incision made, and the upper maxilla of right side was extirpated. Paralysis of the face followed, on account of the division of portio dura nerve. The parts united well; the patient's general health improved materially, and he was discharged.

E. L., age 17.—Has a very rare congenital growth. An elephantiasis surrounded by hair; a pendulous tumor on left knee, flabby, and the hair grows long and thick around the entire surface of the tumor, Removed, and after the union was complete patient was discharged, cured.

R. I., age 30.—Enlargement of the lymphatics, with slight softening; large swelling in parotid region in front of the left ear, below the angle of the jaw, and extending as far up as the zygoma. For five years patient has been thus affected. An incision was made directly over the centre of the tumor in nearly vertical direction, and the entire tumor was removed. Patient discharged, well.

M. M., age 42.—Has had a schirrus removed from left mammary gland three times, and now a fourth growth has appeared over old cicatrix. Tumor removed.

G. C., age 38.—Syphilitic disease of left testicle. An incision was made from the external ring, bringing it down on the inside. As soon as the knife was introduced, a large stream of water gushed out. The spermatic cord was tied with a silver ligature. The patient lost but a very small amount of blood. He was placed upon

℞ *Hydrarg. Bichlor*, gr. $\frac{1}{12}$

*Sodii Iodid*, gr. vi.

three times a day, and did well. Twenty-seven days after the operation he was discharged.

J. M., age 7.—Stricture of æsophagus. Drank some lye about five months ago, and since that time has been unable to swallow without much difficulty. A bougie was inserted, and constriction was found to be very narrow. Bougies of various sizes were introduced, from day to day, for three weeks, at the end of which time patient was discharged, cured.

Yours, E. R. H.

---

SADORUS, Champaign Co., Ill., April 28, 1868.

DR. J. A. ALLEN — *Sir* :—In one No. of the CHICAGO MEDICAL JOURNAL for 1864, I saw an article from you, on cerebro-spinal meningitis, or as it is commonly called, spotted fever. There is, and has been, some of the same prevailing in this locality.

In all the cases I have seen, it is ushered in by a chill, or shivering, with severe pain in the head, or some part of the body, or one of the limbs.

In most cases, the head is drawn back, with extreme sensitiveness of back bart of the neck and spine.

The extremities are very cold, and I find great difficulty in getting up reaction.

In some cases, vomiting is very obstinate ; water scanty, with strangury.

Pulse weak, and often slower than in health.

In most of the cases I find the pupil of the eye dilated, but in one bad case I had it was normal.

In all the cases I have met with, I find the patient very sore to the touch, or motion, and it is sometimes the case that one side is more so than the other. In two cases treated in one house, one, a child of four years, I found the left side the most sensitive, and in the woman it was the reverse.

In two cases, the left side of each was paralyzed; one a boy four years old, the other an adult woman. In the boy, the breathing was spasmodic, and you could see that one side of the chest expanded to an unnatural size, while the other remained stationary. Neither could I detect that any air passed into the lung.

Most of the cases are delirious, but not of an active form.

In the worst attacks reactions do not take place, but the poor sufferers soon sink into a profound coma, and death comes to their relief.

Usually an eruption makes its appearance (sooner or later), of a singular character. It does not look like measles, as one writer has said (in JOURNAL of 1863). I found by pressing on them that they nearly disappeared. It comes nearer resembling purpura, than measles or bullæ. I look upon it as a disintegrated condition of the blood.

The cases that have fallen under my care have led me to think it a disease of the blood (or a poisoned condition of the vital fluid, with a peculiar affinity for the membranes of the brain and spine).

Nor is this strange, when we take in consideration the cerebro-spinal centre.

It is not an acute disease, but sub-acute (at least, I have so considered it.)

It is a very deceptive disease, leading nurses, and, I fear, too frequently, physicians, to think the danger passed, when in reality I look upou that deceptive remission as a bad omen.

I have used the following treatment: *Capsicum* in full doses, *Opi* in moderate, for its stimulant effect; *Camph.; Tinc. of Canthar.;* tonic doses of *Quinine*, and (the cases are in a malarial district) *Tinc. Iron.* On account of the sedative action of alcohol, I have been afraid to use it, and in the use of the above I have in all cases given for the effect, and not for quantity. External, *Rubefacients*, with artificial heat, with due care to its effect, I find useful. Stimulants, internal and external, I find the best. Strong irritants to the spine and extremities. When the surface is dry, I find I can get up an action quicker by the use of hot water, used for a short time, than any thing else.

I have the part dried, and follow by strong rubefacients.

When the surface is moist, I use friction with the hand, and the strongest external stimulants. It is frequently the case that the patient will change from moist to dry several times during the day.

I see nearly all the writers call it cerebro-spinal meningitis. I must enter a protest to the name.

In Watson's practice, a note added by Dr. Condie, it is called typhoid pneumonia.

In most cases I notice a rapid disposition to dissolution of the blood.

I have not written this for publication, but to ask you, by private letter or through the JOURNAL, to give me all you can that is new in the way of treatment. In one bad case of spasms, I did not have any *Chloroform*, and nine miles from home. I used *Bromide of potassium*, which controlled them before the messenger returned with the *Chloroform*, and I did not use it.

Having been a careful reader of your valuable journal for the last four years, and having a high regard for your opinion, I have taken the liberty to ask of you a little time in the consideration of the article, and if not asking of you too much, will be pleased to hear from you on the same, through the Journal.

This makes the fourth time that this disease has made its appearance in that locality. So far I have noticed that the weather has been much the same.

It has so far always been in the fall, winter, or spring, in sudden changes from cold to warm, accompanied with wet. As the article was not written for publication, I have not taken the trouble to give all the symptoms, but have, I think, given sufficient to satisfy you that the disease under consideration is the spotted fever. Excuse me, my dear Dr., for thus intruding on your valuable time. A sincere desire for knowledge is my excuse. A. CATRON.

---

*To the Editor of the Chicago Medical Journal :*

DEAR PROF. ALLEN :—In the editorial observations concerning the report of the case of tetanus, treated by Dr. Baxter, you are correct in the statement that Prof. Gunn suggested the use of the *Calabar bean.* Dr. B. mentioned to me that Prof. Gunn did so. However, as I drew up the report hurriedly, from the notes taken at the time, and was even so uncourteous towards you as not to have copied it before laying it before you, I wish to have it understood that no injustice was meant to be done the learned Professor. The omission was as unintentional on my part as it is unsatisfactory. I may also mention that Drs. Hunt, Edwards, and McDonold assisted at the amputation. Yours truly,

P. CURRAN.

